# Sustainable customer retention through social media marketing activities using hybrid SEM-neural network approach

**DOI:** 10.1371/journal.pone.0264899

**Published:** 2022-03-04

**Authors:** Qing Yang, Naeem Hayat, Abdullah Al Mamun, Zafir Khan Mohamed Makhbul, Noor Raihani Zainol

**Affiliations:** 1 UCSI Graduate Business School, UCSI University, Cheras, Kuala Lumpur, Malaysia; 2 Faculty of Entrepreneurship and Business, Universiti Malaysia Kelantan, Pengkalan Chepa, Kota Bharu, Kelantan, Malaysia; 3 UKM-Graduate School of Business, Universiti Kebangsaan Malaysia, Kuala Lumpur, Selangor Darul Ehsan, Malaysia; University of Oklahama Norman Campus: The University of Oklahoma, UNITED STATES

## Abstract

Social media has changed the marketing phenomenon, as firms use social media to inform, impress, and retain the existing consumers. Social media marketing empowers business firms to generate perceived brand equity activities and build the notion among consumers to continue using the firms’ products and services. The current exploratory study aimed to examine the effects of social media marketing activities on brand equity (brand awareness and brand image) and repurchase intention of high-tech products among Chinese consumers. The study used a cross-sectional design, and the final analysis was performed on 477 valid responses that were collected through an online survey. Partial least squares structural equation modelling (PLS-SEM) and artificial neural network (ANN) analysis were performed. The obtained results revealed positive and significant effects of trendiness, interaction, and word of mouth on brand awareness. Customisation, trendiness, interaction, and word of mouth were found to positively affect brand image. Brand awareness and brand image were found to affect repurchase intention. The results of multilayer ANN analysis suggested trendiness as the most notable factor in developing brand awareness and brand image. Brand awareness was found to be an influential factor that nurtures repurchase intention. The study’s results confirmed the relevance of social media marketing activities in predicting brand equity and brand loyalty by repurchase intention. Marketing professionals need to concentrate on entertainment and customisation aspects of social media marketing that can help to achieve brand awareness and image. The limitations of study and future research opportunities are presented at the end of this article.

## 1. Introduction

By definition, social media encompass online applications, platforms, and media promoting interaction, collaboration, and content sharing among users [1]. The associated marketing component renders it highly convenient to spread information due to its synergy-inducing scale [2], thus highlighting social media as a practical choice for such purpose. In fact, effective information dissemination is a crucial factor in ensuring the success of social media marketing [3]. In 2018, Chinese social media users increased by 100 million [4], while the beginning of 2020 recorded an increment for global social media users amounting to 3.8 billion. Simultaneously, recent data has shown that more than 1 billion people in China employ social media, as reflected by its social media popularising rate of 74% [5]. In line with this, the Global Web Index has reported that a user typically makes use of social media for up to two hours and 42 minutes per day [6]. Therefore, the last decade has underlined social media marketing as an essential marketing tool, emerging as a mainstream research aspect [7].

In general, social media offer consumers a new platform for understanding a product and interacting with people anywhere globally to share product-related experiences [8, 9]. This population is typically embedded with different awareness orientations before making a purchase decision, which can be divided into brand awareness and value awareness [10]. To compare: consumers having the brand awareness orientation perceive the brand as a symbol of credibility and prestige, whereas those with value awareness usually check and compare the prices and quality of different brands through social media to ensure a best-value purchase [11]. To this end, many companies employ social media in carrying out low-cost and high-efficiency marketing activities for consumers [7, 12]. Social media is a marketing tool wielded for four primary purposes: market research and feedback; brand promotion and reputation management; customer service and customer relationship management; and business network [13, 14]. Despite its active incorporation in companies to increase visibility and gain more customers, social media-focused customer loyalty-building and strengthening remain a less-explored area [13, 15]. Therefore, understanding how social media activities affect customer loyalty is essential for enhanced marketing strategies.

The current study discussed the impact of social media-based marketing on brand loyalty through brand equity. Entrepreneurial and large business firms actively engage with social media-based marketing activities to inform and build attractiveness for their prospective and current consumers [16]. Furthermore, the study distinctively highlighted the impact of social media-based marking activities on consumer-level brand equity and brand loyalty in terms of repurchase intention.

The purpose of this article is to systematically and comprehensively examine the impact yielded by social media marketing activities (SMMA) towards the creation of consumer brand awareness and brand image. Accordingly, the research goal denotes filling the gaps identified in previous efforts, which include: (1) evaluating the effect of the components of SMMA (i.e., entertainment, customisation, trends, interaction, and word of mouth) on brand awareness and image; and (2) exploring the impact of SMMA on Chinese consumer repurchase intention through brand awareness and brand image of high-tech products.

## 2. Literature review

### 2.1 Theoretical foundation

Russel and Mehrabian initially conceived the pioneering Stimulus-Organism-Response (SOR) model in early 1974, which underlines the following operating principle: environmental stimuli (S) lead to emotional responses (O), thereby promoting behavioral responses (R). Subsequently, countless retail scholarships have utilised the SOR model to illustrate its importance in retailing [17, 18]. The model is currently widely implemented in consumer behavior studies [19–22]. Based on previous studies, SMMA plays a vital role in influencing customer level of brand awareness and brand image. Accordingly, the SOR model offers a structured method for evaluating the impact of perceived SMMA on brand equity and repurchase intention [11, 23–25].

The SOR model is selected as the research model for the current study for three specific reasons. First, previous studies have comprehensively implemented the SOR framework to study human-computer interaction leading to consumer buying behavior [26–28]. Secondly, the SOR theory provides the direction of investigations undertaken in the hotel management, food delivery, and other services industries [29–32]. Finally, a scant amount of literature is available pertaining to consumer behavior in China despite the model’s extensive implementation across different countries in assessing the particular topic.

#### 2.1.1. SMMA recognised as an environmental stimulus

Previous research efforts have detailed the role played by SMMA to aid in creating value, enhancing brand awareness, and building customer relationships [23–25]. Furthermore, some studies have particularly emphasised its use as a marketing stimulus towards enhancing the customer shopping experience and influencing purchase behavior. For example, Zhang and Benyoucef’s [22] research has supported SMMA as an environmental stimulus in the SOR model. In contrast, social media is thought to play an important role when companies build relationships with their customers via marketing activities [24]. Similarly, Kim and Ko [33] have differentiated its characteristics into five categories, namely: entertainment, interaction, fashion, customisation, and WOM, which are then applied to luxury brands. Subsequently, previous works led to this research defining SMMA components as entertainment, interactivity, popularity, customisation, and perceived risk accordingly.

*Entertainment* is the result of fun and entertainment in using social media [34]. Thus, the entertainment component of social media is deemed essential, whereby it enriches positive emotions and generates behaviours that involve purchase intentions [14]. *Interaction* is the exchange of opinions and ideas occurring between social media and consumers. A stronger interaction on social media allows consumers a deeper understanding of brand content and empowers better brand comprehension of user ideas and preferences, thereby contributing to the brand’s social media platform itself [24]. Concurrently, consumers can also exchange realised social media platform experiences [14], whereas social media user-generated content (UGC) has emerged as an alternative brand-customer interaction [24, 35]. *Trendiness* is crucial and otherwise defined using the term ‘trends,’ which details the provision of the latest information pertaining to any products or services [25]. Tangentially, Valaei and Nikhashemi’s [36] research has underlined brand style and price as particularly notable factors determining Generation Y consumers’ willingness to buy fashion items. Therefore, the trends and styles positioned by brands can attract more consumers of the younger age range due to their likings for new trends and trendy brands [37]. *Customisation* is defined as the degree to which a brand provides specific services to meet the unique tastes and needs exhibited by consumers [38]. During the consumption process, most customers still want to obtain specific services. Therefore, the current research describes survey personalisation as customer perception of social media in providing customised services and meeting their preferences. Accordingly, brands can provide private and customised experiences tailored to each customer based on personalised portals and offline shops to improve further their brand image and brand loyalty [39]. Besides, personalisation will accurately help customers locate the products they require, thus indirectly promoting the purchases [40]. *Word of mouth (WOM)* is the most natural and common phenomenon encountered in consumer behavior [22]. It can denote a series of communication activities carried out by a company or product, which is usually regarded as non-commercial and private [41]. Similarly, WOM is also a source of information in the purchase decision process in which consumers will consider product performance, changes before and after purchase, and consequences of the purchase decision [42, 43]. The more familiar and trustworthy the WOM information sources are, the more significant their impact on purchasing decisions [44]. WOM is more effective than alternative SMMA channels in influencing consumer decision-making [45].

#### 2.1.2. Brand equity recognised as customer emotional response

In general, brand equity is defined as intangible assets related to brand names and brand symbols due to the possible effect of brand preference on the brand value as perceived by brand consumers [46]. Keller [47] has classified it into brand awareness and brand image, thereby describing brand equity as a social and cultural phenomenon. Meanwhile, brand image is firmly embedded in consumer minds and denotes the associated symbolic meaning, which the brand pursues [48]. By definition, brand image denotes the impression held by a brand in consumer memory, thus categorised into deep, general, and vague impressions accordingly [47]. The brand image helps understand and accept the brand’s meaning through consumer perception [49], which is a collective result of various marketing activities and consumer experience [50]. In contrast, brand awareness refers to consumers’ ability to recognise or remember a brand [50], which aids them in searching for products to be purchased faster [51]. This element typically includes four levels, namely: brand recognition, brand recall, top of the mind brand, and dominant brand [52].

#### 2.1.3. Repurchase intention recognised as consumer response

Consumer response makes up the final part of the SOR model [28], which can be divided into two situations: response and avoidance. Here, the response behavior depicts customer willingness to purchase a product and positive WOM, whereas the avoidance behavior denotes opposite or negative WOM and unwilling purchase [20]. These responses underpin the current work’s investigation on Repurchase intention, which is otherwise characterised as consumer repurchase intention. Achieving customer loyalty is typically known as the most significant objective of marketing activities as the element is attributed to satisfied customers and consistent sales [33]. Therefore, repurchase intention is the key to fostering the relationship between customers and brands, whereby some studies have pinpointed increased loyalty and its correlated effect on reduced marketing costs and increased sales [53]. A brand owner may find it highly necessary to adjust its marketing strategy to retain valuable consumers and increase repurchase intention [25].

### 2.2 Repurchase intention

Repurchase intention generally refers to customer judgement of a specific brand product [54]. Alternatively, brand loyalty describes customer recognition of a particular brand, chosen among many brands as bolstered by the willingness to buy and repurchase products or services [55]. Therefore, brand loyalty is perceived as the repurchase intention, thus directly reflecting consumer thoughts when choosing to repurchase a particular brand [54]. Furthermore, brand loyalty is commonly expressed as the consumer tendency to purchase or repurchase brand-related products [56, 57]. However, the level of attractiveness shown by their alternatives may affect the relationship between recovery satisfaction and repurchase intentions [58]. Here, elements influencing consumer repurchase intentions vary, including the lenient return policy and perceived fairness of return experience on top of the common return issues [59]. Besides, loyal brand users have low price sensitivity to associated brand products, which are also introduced to their friends. Therefore, these positive sharing behaviours allow many potential customers to the brand, increasing initial and second purchase intention [60].

### 2.3. Social media marketing activities and brand awareness

#### 2.3.1 Entertainment and brand awareness

Entertainment generally refers to the fun aspect embedded in brand marketing content, which has been underlined by Kim and Ko [33] and Seo and Park [24] as an integral part of SMMA. Today, brand products are no longer tethered to traditional displays; instead, they are integrated with entertainment components to establish a stronger emotional connection with consumers [61]. Furthermore, it has been pinpointed as a factor that directly affects consumer attitudes towards brands [62], whereby Bilgin [63] has specifically detailed its significant effect on brand awareness and brand image. Accordingly, improving brand awareness is among the well-known corporate SMMA [33, 64]. For example, Seo and Park [24] have pointed out that such activities performed by aviation and hotel businesses positively impact brand awareness and brand image. As such, the following hypotheses are formulated:

H1a: *Entertainment positively affects brand awareness among Chinese consumers*.

H1b: *Entertainment positively affects brand image among Chinese consumers*.

#### 2.3.2 Customization and brand equity

Consumers typically believe that personalised brand recommendations align with their product preferences, and their more personalised needs to a higher degree [65]. In social media, customisation refers to the target audience of a message. Zhu and Chen [66] have thus identified the two types of publishing, depending on the level of message customisation: custom message and broadcast [56]. Here, customised information targets specific people or a small number of audiences (e.g., Facebook posts), whereas a broadcast generally contains messages directed at anyone interested in the content material (e.g., Twitter tweets). Therefore, the following hypotheses are proposed:

H2a: *Customisation positively affects brand awareness among Chinese consumers*.

H2b: *Customisation positively affects brand image among Chinese consumers*.

#### 2.3.3 Trendiness and brand equity

Technology empowers firms to cultivate trends and enrich customer satisfaction and experience [67]. Trends are known for their significant impact on customer brand equity, especially in the context of young consumers [68]. Trendiness depicts the firms’ ability to foster and spread pertinent information that empowers their brand equity [8]. The adoption of social media to attract consumers has increased among SMEs [16]. In the luxury goods industry, fashion trends denote an essential element in SMMA and positively impact brand equity [60]. Kim and Lee [67] claimed that social media-based trendiness spurs brand awareness and brand image among the prospective consumers. Similarly, social media trends offer extensive awareness among users and help develop the brand image. Therefore, the following hypotheses are developed:

H3a: *Trendiness positively affects brand awareness among Chinese consumers*.

H3b: *Trendiness positively affects brand image among Chinese consumers*.

#### 2.3.4 Interaction and brand equity

Interaction mainly describes the dynamic communication between enterprises and consumers [35], and social media empowers both to interact facilitatively. Social media-based interaction simplifies the brand communication to the brand consumers and nurtures consumers’ brand experience and satisfaction [69]. Consumers and brands communicate and interact using various social media platforms [23]. Such platforms can be utilised to build consumer brand awareness and establish the right brand image concurrently [64]. Wang et al. [59] documented that social media-based interaction influences brand awareness and brand image building among young fashion retail consumers. Therefore, the following hypotheses are formulated:

H4a: *Interaction positively affects brand awareness among Chinese consumers*.

H4b: *Interaction positively affects brand image among Chinese consumers*.

#### 2.3.5 Word of mouth and brand equity

In general, word of mouth refers to consumer perception regarding the degree to which other customers recommend and share the latter’s social media experiences [13]. Consumers like to share their positive or negative experiences on social media [67]. An industry survey has revealed that a whopping 91% of respondents would consider online reviews, ratings, etc., prior to any product purchases from e-commerce sites. In contrast, nearly 46% agree that such reviews influence their purchasing decisions [70]. Consumers’ comments on social media facilitate prospective consumers’ awareness and help build the brand image that later influences purchase intention [44]. Chahal, Wirtz, and Verma [71] claimed that online brand reputation directly affects perceived brand equity among consumers. Kim and Lee [67] suggested the positive influence of word of mouth on the levels of brand awareness and brand image among young Bangladeshi consumers. As such, the following hypotheses are proposed:

H5a: *Word of mouth positively affects brand awareness among Chinese consumers*.

H5b: *Word of mouth positively affects brand image among Chinese consumers*.

### 2.4 Brand equity and repurchase intention

Theoretically, brand equity is a subjective assessment of consumer brand preferences [10], whereby a brand rated as unique and appropriate by consumers indicates a high level of brand equity perceived for it [32]. Accordingly, a positive brand attitude can positively influence the customers’ repurchase intentions [55]. Zhang et al. [21] have postulated the positive link between brand equity and customer loyalty. In particular, brand equity based on brand awareness and brand image positively influence repurchase intention. For instance, brand awareness and brand image promote brand purchase and repurchase [10]. Thus, the following hypotheses are generated:

H6a: *Brand awareness positively affects repurchase intention among Chinese consumers*.

H6b: *Brand image positively affects repurchase intention among Chinese consumers*.

### 2.5 Mediating effect of brand equity

The impact of SMMA on brand equity has been confirmed in multiple previous studies in which the latter is considered the reason or motivation for purchasing certain brands. Therefore, higher brand equity can be correlated with higher robustness for consumer preference and willingness to buy any products [72, 73]. Alternatively, brand awareness also affects consumer attitudes towards brands, further stimulating their brand choice [64]. In this matter, Keller [47] believes that regardless of the attributes being related to specific brand products or not, they vigorously promote the formation of brand associations, which will, in turn, directly affect consumer purchase or repurchase intentions. Therefore, brand loyalty is considered a necessary factor for ensuring repeat purchases [72, 74, 75]. Hence, the following hypothesis is generated:

Hypothesis (HM): *Brand awareness and brand image mediate the relationship between entertainment*, *customisation*, *trendiness*, *interaction*, *and word of mouth on the repurchase intention among Chinese consumers*.

## 3. Research methodology

### 3.1 Research design

**Fig 1:**
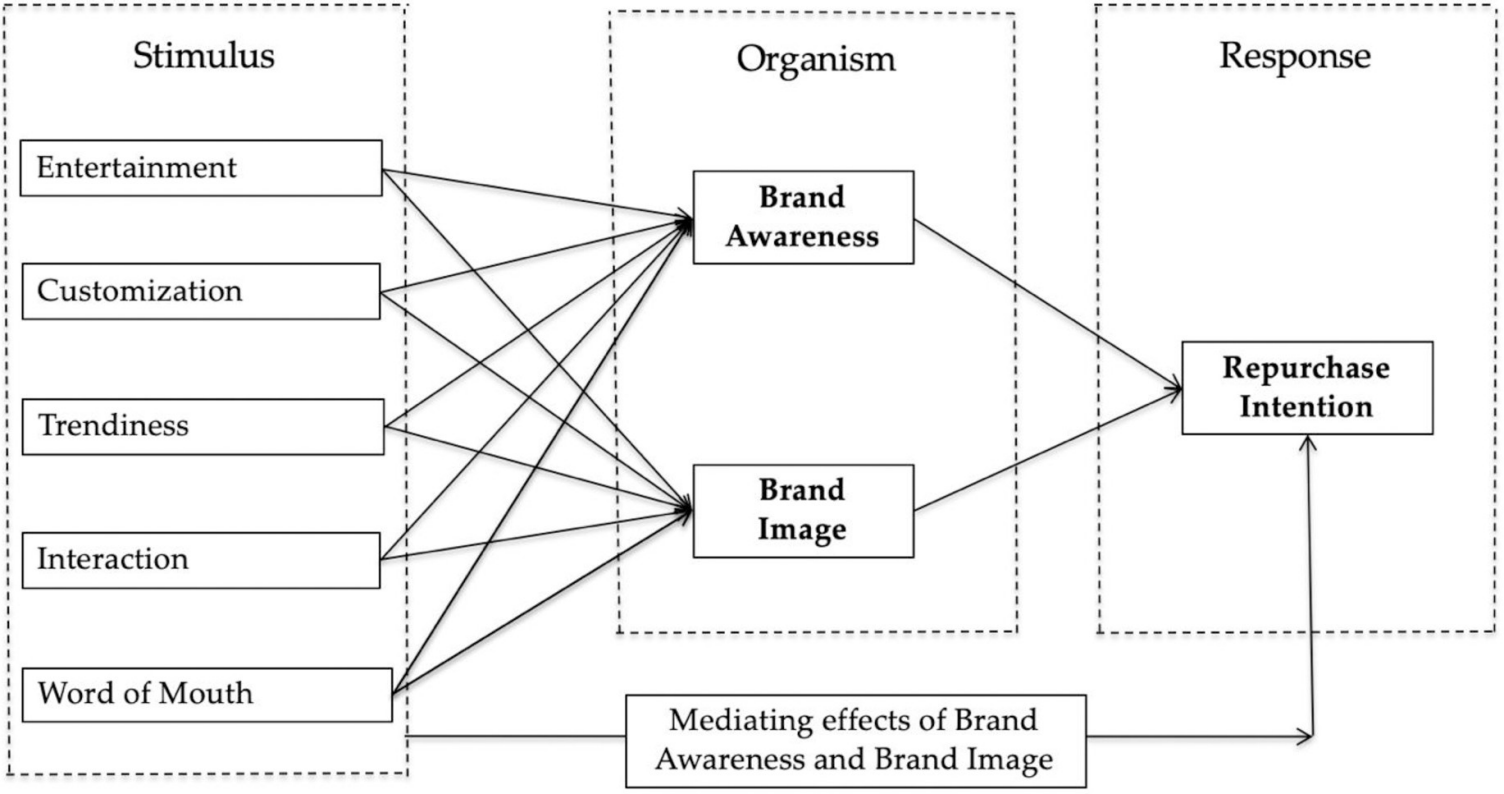
Research framework.

The current study aimed to measure the impact of SMMA on brand awareness and brand image, thereby leading to the repurchase of high-tech products from a brand among Chinese consumers. The hypotheses and associations are thus designed and tested according to Fig 1. The explanatory research design was implemented to depict the relationship between tested variables and determine its cause and impact [76]. Concurrently, quantitative methods were incorporated to study the relationship between variables, whereby the cross-sectional survey method was utilised for data collection purposes. Gathered from the target population, this allowed an exploration of the phenomenon being studied in work.

### 3.2 Sample selection and data collection method

The current study targeted the general population living in China who use social media platforms, namely WeChat, Tencent QQ, Sina Weibo, Youku Tudou, and Douyin, which are used by firms to promote their products. Therefore, the target population of this study included a total of 999.95 million social media users in China [77]. The sample size calculation was performed using G-Power 3.1 software. With power of 0.95, effect size of 0.15, and a total of seven predictors, the required sample size for the current study was 168 [78]. Moreover, the minimum sample of 200 is suggested for PLS-SEM [79]. The study aimed to employ the second-generation statistical analysis technique of structural equation modelling; therefore, the study collected data from more than 400 respondents. As the study was conducted during the COVID-19 lockdown, online survey was opted to protect the respondents and surveyors from COVID-19. The survey was conducted online (http://www.wjx.cn/) from May 2020 to June 2020.

Local ethics committees (Universiti Malaysia Kelantan, Malaysia) ruled that no formal ethics approval was required in this particular case based on the following reasons: (1) this study did not collect any medical information; (2) there was no known risk involved; (3) this study did not intend to publish any personal information; (4) this study did not collect data from underaged respondents. Moreover, this study was performed in accordance with the Declaration of Helsinki. Written informed consent for participation was obtained from all survey respondents. The respondents were required to read and provide their agreement to the following ethical statement posted at the start of the survey before they were allowed to proceed to answering the survey questions: “*There is no compensation for responding nor is there any known risk*. *In order to ensure that all information will remain confidential*, *please do not include your name*. *Participation is strictly voluntary and you may refuse to participate at any time*”. No data was collected from anyone under 18 years old.

### 3.3 Survey instrument

The questionnaire consisted of two parts, namely Parts A and B. First, Part A included question items about the population profile, gender, age, monthly income, and education level. Meanwhile, Part B comprised question items about SMMA, brand equity, and consumer loyalty. In total, 34 measurement items were employed to estimate SMMA, brand awareness, brand image, and repurchase intention. The measurements were subsequently evaluated using a 5-point Likert scale ranging from 1 (Strongly disagree) to 5 (Strongly agree). A list of the question items and sources for implemented scales is detailed in Table 1 accordingly.

**Table 1:** Survey instrument.

Code	Questions	Source
ENT–Item 1	It is fun to use high-tech brand’s social media.	Kim & Ko [80]
ENT–Item 2	It is interesting of the contents shown in the high-tech brand’s social media.	
ENT–Item 3	It is exciting to use high-tech brand’s social media.	
ENT–Item 4	It is easy to kill time using high-tech brand’s social media.	
ENT–Item 5	It is fun to collect information on brands or items through a high-tech brand’s social media.	
INT–Item 1	It is easy to deliver my opinion through high-tech brand’s social media.	Kim & Ko [80]; Yadav & Rahman [25]
INT–Item 2	It is possible to exchange opinions or conversation with other users through a high-tech brand’s social media.	
INT–Item 3	It is possible to share information with other users through a high-tech brand’s social media.	
INT–Item 4	It is possible for me to share and update the existing content on a high-tech brand’s social media.	
INT–Item 5	It is possible for this high-tech brand’s brand regularly interacts with its followers and fans.	
TRD–Item 1	It is the newest information of contents shown in the high-tech brand’s social media.	Kim & Ko [80]; Yadav & Rahman [25]; Ladhari et al. [37]
TRD–Item 2	It is very trendy to use high-tech brand’s social media.	
TRD–Item 3	It is available of anything trendy on high-tech brand’s social media.	
TRD–Item 4	High-tech products are one of the most important ways to express my individuality.	
CUT–Item 1	High-tech brand’s social media provides customized service.	Kim & Ko [80]; Kim & Ko [33]
CUT–Item 2	It is possible to search customized information on high-tech brand’s’ social media.	
CUT–Item 3	It is possible to provide lively feed information I am interested in on high-tech brand’s social media.	
CUT–Item 4	It is easy to use high-tech brand’s social media.	
WOM–Item 1	It is possible for me to share opinions on brands, items, or services acquired from a high-tech brand’s social media with my acquaintances.	Seo & Park [24]; Hutter et al. [81]; Kim & Ko [80]
WOM–Item 2	It is possible for me to win my friends and relatives as high-tech brand’s fans.	
WOM–Item 3	It is fun for me to inspire others about high-tech brand’s products.	
WOM–Item 4	I will recommend this high-tech brand’s social media.	
BAW–Item 1	I am always aware of this high-tech brand’s change in production.	Hutter et al. [81]; Godey et al. [64]
BAW–Item 2	I have no difficulties to remember this high-tech brand.	
BAW–Item 3	I know all the high-tech brand’s models.	
BAW–Item 4	I can distinguish the different high-tech brand’s mode.	
BIM–Item 1	This high-tech brand is a leader in the industry.	Sasmita & Mohd Suki [82]; Bilgin [63]; Seo & Park [24]
BIM–Item 2	This high-tech brand is customer-cantered.	
BIM–Item 3	I have fond memories regarding this high-tech brand.	
BIM–Item 4	This particular product/brand has a differentiated image in comparison with the other product/brand.	
RPI–Item 1	I will consider this brand first when I want to buy tech gadgets.	Athapaththu & Kulathunga [83]
RPI–Item 2	I would be comfortable shopping at this brand.	
RPI–Item 3	I intend to continue using this brand in the future.	
RPI–Item 4	I would like to buy new products/services from this brand.	

Note: EN: Entertainment; CU: Customization; TR: Trendiness; IN: Interaction; WM: Word of Mouth; BA: Brand Awareness; BI: Brand Image; RI: Repurchase Intention.

### 3.4 Preliminary data preparation and multivariate normality

The outlier analysis with the Mahalanobis distance (D2) measure was conducted to estimate multivariate outliers [79]. Multiple regression analysis engaged all input variables on the outcome construct and saved the Mahalanobis distance for all cases. Cases with D2 of more than 26.125 were declared outliers; as a result, 35 cases were dropped. The subsequent analysis was performed with only 477 valid cases. Peng and Lai [84] have cautioned against making general statements about the partial least squares (PLS) estimation model capability as the action may violate the typical multivariate assumption despite the model not requiring a multivariate normal data distribution. Therefore, this study employed the Smart-PLS online tool to test the multivariate normality. The calculations carried out revealed p-values less than 0.5 for the multivariate skewness and kurtosis for Mardias’ coefficient. These outcomes successfully confirmed the non-normality of the data.

### 3.5 Common Method Bias (CMB)

As suggested by Podsakoff, Mackenize, and Podsakoff [85], CMB was evaluated based on the results of Harman single factor analysis, which served as a diagnostic tool in this study. The obtained results suggested that a single factor accounted for 35.19%, which was less than the prescribed limit of 50%. In other words, CMB was not a critical issue for the current study [85]. Moreover, Kock [86] recommended performing a full collinearity test to gauge the CMB issue. A common variable formed, and all the variables regressed on the common variable as an outcome variable. The variance inflation factor (VIF) for entertainment (1.685), customisation (2.063), Interaction (3.451), trendiness (2.948), word of mouth (2.202), brand awareness (3.433), brand image (2.277), and repurchase intention (2.781) did not exceed 5. This reaffirmed that CMB was not a severe issue for the current study [86]. The correlations matrix of the latent variables also showed that CMB was not an issue, as all correlations did not exceed 0.900 [85].

### 3.6 Data analysis method

Partial Least Squares—Structural Equation Modelling (PLS-SEM) is driven by maximising the interpretation variance of related latent structures [79]. It was implemented in this study to explore the impact of SMMA on Chinese consumer repurchase intentions in the presence of non-normality issues. Artificial neural network (ANN) analysis is a non-compensatory analytical technique with deep learning algorithms based on three layers: input, output, and hidden layers. The hidden layer connects the input neurons with the output neurons [87], acting as the block-box similar to the human brain [88]. The data are divided into three parts for training, testing, and holding out part of the sample. The study utilized the Root Mean Square Errors (RMSE) value of trained and tested data to identify the predictive accuracy [89, 90].

## 4. Data analysis

### 4.1 Demographic characteristics of respondents

As presented in Table 2, the majority of the respondents in this study were female (59.7%), while the remaining 40.3% were men. The respondents were grouped into the following age groups: (1) 18–26 years (46.3%); (2) 27–34 years (22.8%); (3) 35–42 years (13.8%); (4) 43 years and above (16.9%). Furthermore, 34.5% of the total respondents recorded monthly income of less than 3,000 yuan, followed by those with monthly income of between 3,001 and 6,000 yuan (30.6%), monthly income of between 6,001 and 9,000 yuan (15.3%), and lastly, monthly income of more than 9,000 yuan (19.4%). Among 477 respondents, only 16.4% consisted of high school students. The majority of the respondents (63.3%) were undergraduates, followed by graduate students (20.5%). In terms of the usage of electronic gadgets, the majority of the respondents (43.1%) used zero to two types, followed by those who used three to five types (33.9%). About 22.8% of the total respondents reported using more than six types. It should be noted that this study was not limited to any specific product brand or category, but rather a general assessment of high-tech products collectively.

**Table 2:** Demographic characteristics.

	N	%		N	%
Gender			Education		
Male	192	40.3	High school certificate	77	16.4
Female	285	59.7	Bachelor degree or equivalent	302	63.3
Total	477	100.0	Master’s degree and Above	98	20.5
			Total	477	100.0
Age Group					
18–26 years	221	46.3	Usage of Electronic Gadgets		
27–34 years	109	22.8	(Smart Phones, Tablet, Power Bank, Earphone, Projector, Intelligent Sweeping Robot, Drone)		
35–42 years	81	13.8			
43 years and Above	81	16.9	0–2	206	43.1
Total	477	100.0	3–5	162	33.9
			6–8	109	22.8
Average Monthly Income (Yuan)			Total	477	100.0
Below 3000	165	34.5			
3001 to 6000	146	30.6			
6001 to 9000	73	15.3			
More than 9000	93	19.4			
Total	477	100.0			

### 4.2 Reliability and validity

Reliability refers to the consistency shown by the measurement items according to the results obtained via the measurement tools implemented, thereby objectively reflecting the reasonable degree of the measured characteristics. In contrast, validity denotes their effectiveness by measuring whether the comprehensive evaluation system can accurately reflect the evaluation purposes and requirements. Thus, it echoes the measurement of feature accuracy in measuring by using the measuring tool.

Table 3 details the descriptive statistics, validly, and reliability criteria employed to evaluate the items used in the study. However, in reliability analysis, the Cronbach’s alpha (CA) coefficient size was assessed in measuring the questionnaire reliability. In general, a coefficient larger than 0.9 indicates excellent reliability, while a coefficient above 0.8 is good. Meanwhile, values between 0.5 and 0.9 reflect a reasonable outcome, whereas coefficients lower than 0.5 render the outcomes non-trustworthy. Accordingly, CA values shown in Table 3 reveal that all variables generated values greater than 0.8, indicating the latent constructs’ reliabilities.

**Table 3:** Reliability and validity.

Variables	No. Items	Mean	Standard Deviation	Cronbach’s Alpha	Dijkstra-Hensele’s *rho*	Composite Reliability	Average Variance Extracted	Variance Inflation Factors
ENT	5	3.815	0.916	0.828	0.830	0.879	0.593	1.720
CUT	5	3.756	0.861	0.868	0.869	0.904	0.653	1.594
TDR	4	3.754	0.857	0.807	0.807	0.866	0.564	2.039
INT	4	3.852	0.906	0.841	0.843	0.888	0.613	2.685
WOM	4	3.774	0.932	0.815	0.824	0.871	0.575	1.765
BAW	4	3.751	0.973	0.818	0.820	0.873	0.580	1.717
BIM	4	3.772	0.968	0.802	0.805	0.864	0.560	1.717
RPI	4	3.810	0.935	0.854	0.856	0.896	0.632	-

Note: ENT: Entertainment; CUT: Customization; TDR: Trendiness; INT: Interaction; WOM: Word of Mouth; BAW: Brand Awareness; BIM: Brand Image; RPI: Repurchase Intention.

Source: Author’s data analysis.

Moreover, Dillon Goldstein rho (DG rho) values more than 0.80 as seen for all variables, further confirming the measurement item reliability. We also utilized the composite reliability (CR), and the CR score for all the study constructs are well above 0.85, showing satisfactory reliabilities. Table 3 depicts the acceptable convergent validity attained by the constructs due to values higher than 0.50. According to the recommendation, convergent validity was obtained if the average variance extracted (AVE) value is higher than 0.50. Finally, testing for multicollinearity issues was performed by assessing the variance inflation factors (VIF). The VIF value of each factor is less than 5, suggesting that no major collinearity problem was present. From the reliability and validity testing undertaken, the Composite Reliability (CR) and Average Variance Extracted (AVE) for each factor were relatively good, indicating relatively good data validity.

### 4.3 Discriminant validity

For the current study, Fornell-Larcker criterion, heterotrait-monotrait (HTMT) ratio of correlations, as well as loadings and cross-loadings were used for the evaluation of discriminant validity. As for the estimation of Fornell-Larcker criterion, the square root of AVE of the construct must be greater than the corresponding correlation coefficient in order to establish discriminant validity. The obtained results in Table 4 showed that the study’s constructs showed suitable discriminant validity. Following that, the HTMT ratio served as a tool to estimate discriminant validity [91]. As shown in Table 4, all HTMT ratios did not exceed the threshold value of 0.900, which showed that the study’s latent construct achieved suitable discriminant validity [79]. This study further verified discriminant validity via a comparison between the loadings and cross-loadings of the tested constructs. Generally, loading is the contribution of an item to the latent variable to which it belongs [79], whereas cross-loading is the contribution of an item to other latent variables. The loading of an item that exceeds its cross-loadings indicates that the item contributes more to the latent variable to which it belongs. For the current study, discriminative validity was deemed good. Table 5 shows all loadings and cross-loadings generated, whereby almost all loadings in the current study exceeded 0.7. Besides that, the loadings of all items on their respective corresponding latent variables exceeded their cross-loadings, substantiating the goodness of the questionnaire design and data validity.

**Table 4:** Discriminant validity scores.

	ENT	CUT	TDR	INT	WOM	BAW	BIM	RPI
Fornell-Larcker Criterion								
ENT	0.770							
CUT	0.496	0.808						
TDR	0.571	0.508	0.751					
INT	0.569	0.550	0.661	0.783				
WOM	0.379	0.417	0.486	0.651	0.758			
BAW	0.510	0.445	0.707	0.723	0.554	0.761		
BIM	0.385	0.423	0.563	0.554	0.487	0.646	0.748	
RPI	0.443	0.425	0.622	0.613	0.553	0.709	0.607	0.795
HTMT Ratio								
ENT	-							
CUT	0.583	-						
TDR	0.698	0.607	-					
INT	0.677	0.645	0.803	-				
WOM	0.453	0.492	0.594	0.780	-			
BAW	0.615	0.526	0.870	0.873	0.669	-		
BIM	0.471	0.505	0.699	0.675	0.595	0.795	-	
RPI	0.522	0.492	0.747	0.720	0.653	0.846	0.732	-

**Note:** ENT: Entertainment; CUT: Customization; TDR: Trendiness; INT: Interaction; WOM: Word of Mouth; BAW: Brand Awareness; BIM: Brand Image; RPI: Repurchase Intention.

**Source:** Author’s data analysis.

**Table 5:** Loadings and cross-loading.

Code	ENT	CUT	TDR	INT	WOM	BAW	BIM	PRI
ENT–Item 1	*0*.*766*	0.380	0.415	0.385	0.293	0.368	0.279	0.318
ENT–Item 2	*0*.*800*	0.389	0.475	0.443	0.268	0.412	0.315	0.323
ENT–Item 3	*0*.*798*	0.350	0.445	0.424	0.239	0.346	0.293	0.339
ENT–Item 4	*0*.*747*	0.388	0.459	0.434	0.249	0.371	0.261	0.330
ENT–Item 5	*0*.*736*	0.396	0.403	0.491	0.389	0.450	0.325	0.388
CUT–Item 1	0.433	*0*.*814*	0.448	0.448	0.317	0.345	0.306	0.353
CUT–Item 2	0.464	*0*.*793*	0.437	0.424	0.320	0.379	0.347	0.349
CUT–Item 3	0.368	*0*.*797*	0.377	0.450	0.347	0.319	0.316	0.341
CUT–Item 4	0.375	*0*.*812*	0.398	0.445	0.362	0.371	0.369	0.346
CUT–Item 5	0.366	*0*.*825*	0.395	0.455	0.338	0.377	0.364	0.328
TDR–Item 1	0.393	0.389	*0*.*749*	0.516	0.376	0.518	0.429	0.398
TDR–Item 2	0.430	0.426	*0*.*764*	0.530	0.409	0.530	0.418	0.506
TDR–Item 3	0.407	0.387	*0*.*745*	0.486	0.372	0.539	0.445	0.464
TDR–Item 4	0.491	0.365	*0*.*738*	0.476	0.343	0.541	0.410	0.474
INT–Item 1	0.467	0.432	0.504	*0*.*807*	0.524	0.572	0.470	0.509
INT–Item 2	0.409	0.456	0.505	*0*.*762*	0.513	0.535	0.409	0.412
INT–Item 3	0.454	0.417	0.528	*0*.*827*	0.524	0.593	0.460	0.522
INT–Item 4	0.455	0.414	0.533	*0*.*730*	0.510	0.556	0.398	0.460
WOM–Item 1	0.244	0.258	0.285	0.384	*0*.*669*	0.296	0.268	0.301
WOM–Item 2	0.283	0.346	0.354	0.546	*0*.*792*	0.439	0.400	0.409
WOM–Item 3	0.229	0.340	0.362	0.508	*0*.*791*	0.442	0.398	0.406
WOM–Item 4	0.282	0.319	0.375	0.501	*0*.*783*	0.432	0.386	0.460
BAW–Item 1	0.411	0.377	0.568	0.558	0.435	*0*.*790*	0.518	0.526
BAW–Item 2	0.405	0.354	0.523	0.532	0.405	*0*.*767*	0.485	0.533
BAW–Item 3	0.356	0.309	0.497	0.551	0.394	*0*.*716*	0.448	0.533
BAW–Item 4	0.396	0.337	0.587	0.565	0.448	*0*.*814*	0.566	0.586
BIM–Item 1	0.241	0.274	0.398	0.407	0.359	0.480	*0*.*751*	0.468
BIM–Item 2	0.301	0.322	0.489	0.428	0.362	0.560	*0*.*801*	0.475
BIM–Item 3	0.281	0.361	0.425	0.443	0.396	0.508	*0*.*799*	0.468
BIM–Item 4	0.274	0.311	0.367	0.422	0.385	0.419	*0*.*699*	0.426
RPI–Item 1	0.385	0.341	0.523	0.505	0.479	0.567	0.500	*0*.*808*
RPI–Item 2	0.353	0.352	0.456	0.483	0.425	0.516	0.466	*0*.*753*
RPI–Item 3	0.309	0.305	0.448	0.449	0.402	0.538	0.447	*0*.*815*
RPI–Item 4	0.327	0.301	0.501	0.486	0.442	0.567	0.499	*0*.*830*

**Note:** ENT: Entertainment; CUT: Customization; TDR: Trendiness; INT: Interaction; WOM: Word of Mouth; BAW: Brand Awareness; BIM: Brand Image; RPI: Repurchase Intention.

Source: Author’s data analysis.

### 4.4 Path analysis

Table 6 presents the results of path analysis. The recorded path coefficient for the influence of entertainment on brand awareness (*β* = 0.042, *p* = 0.114) indicated the insignificant but positive influence of entertainment on brand awareness. Thus, H1a was not supported. Meanwhile, the path coefficient for the influence of customisation on brand awareness (*β* = -0.033, *p* = 0.163) revealed that it did not affect brand awareness. Thus, H2a was not statistically supported. Similarly, the path coefficient for the influence of trendiness on brand awareness (*β* = 0.390, *p* = <0.001) displayed its significant effect on brand awareness; thus, offering support to accept H3a. In contrast, the path coefficient for the influence of interactions on brand awareness (*β* = 0.188, *p* < 0.001) indicated its significant and positive influence on brand awareness, which supported H4a. As for the influence of word of mouth on brand awareness, the recorded path coefficient (*β* = 0.109, *p* < 0.001) denoted its significant influence on brand awareness. Thus, H5a was supported.

**Table 6:** Path coefficients.

Hypo		Beta	CI—Min	CI—Max	*t*	*p*		*r* ^2^	*f* ^*2*^	Q^2^	Decision
*SMMA and Brand Awareness*											
H_1a_	ENT -> BAW	0.042	-0.019	0.110	1.064	0.144			0.003		Reject
H_2a_	CUT -> BAW	-0.033	-0.088	0.023	0.983	0.163			0.002		Reject
H_3a_	TRD -> BAW	0.390	0.310	0.467	8.168	<0.001		0.624	0.198	0.355	Accept
H_4a_	INT -> BAW	0.188	0.075	0.297	2.779	<0.001			0.150		Accept
H_5a_	WOM -> BAW	0.109	0.044	0.180	2.601	<0.001			0.018		Accept
*SMMA and Brand Image*											
H_1b_	ENT -> BIM	-0.014	-0.089	0.059	0.309	0.379			0.000		Reject
H_2b_	CUT -> BIM	0.093	0.021	0.171	2.034	0.021			0.009		Accept
H_3b_	TRD -> BIM	0.312	0.206	0.416	4.853	<0.001		0.401	0.080	0.218	Accept
H_4b_	INT -> BIM	0.389	0.299	0.473	7.252	<0.001			0.022		Accept
H_5b_	WOM -> BIM	0.179	0.098	0.262	3.585	<0.001			0.030		Accept
*Brand Equity on Repurchase Intention*											
H_6a_	BAW -> RPI	0.544	0.468	0.617	11.796	<0.001			0.376		Accept
H_6b_	BIM -> RPI	0.255	0.172	0.340	4.957	<0.001		0.541	0.082	0.336	Accept

**Note:** ENT: Entertainment; CUT: Customization; TDR: Trendiness; INT: Interaction; WOM: Word of Mouth; BAW: Brand Awareness; BIM: Brand Image; RPI: Repurchase Intention.

Source: Author’s data analysis.

Meanwhile, the recorded path coefficient for the influence of entertainment on brand image (*β* = -0.014, *p* = 0.379) indicated its non-effect on brand image, rendering the rejection of H1b. Similarly, the recorded path coefficient for the influence of customisation on brand image (*β* = 0.093, *p* = 021) revealed its influence on brand image. Thus, H2b was accepted. In contrast, the recorded path coefficient for the influence of trendiness on brand image (*β* = 0.312, *p* < 0.001) showed its significant and positive influence on brand image. Thus, H3b was accepted. Likewise, the recorded path coefficient for the influence of interaction on brand image (*β* = 0.389, *p* < 0.001) revealed its significant and positive impact, rendering the acceptance of H4b. Additionally, the recorded path coefficient for the influence of word of mouth on brand image (*β* = 0.179, *p* < 0.001) revealed its significant and positive influence. Thus, H5b was supported.

In this study, both H6a and H6b were also accepted. The recorded path coefficients for the effects of brand awareness (*β* = 0.544, *p* < 0.001) and brand image (*β* = 0.255, *p* < 0.001) on repurchase intention indicated their respective significant and positive effects.

### 4.5 Mediation effects

As shown in Table 7, this study employed indirect effect coefficients, confidence intervals, and p-values to measure the mediation effects of brand equity in terms of brand awareness and brand image on SMMAs (in terms of entertainment, customisation, trendiness, interaction, and word of mouth) and repurchase intention. The obtained results revealed that brand awareness (*β* = 0.023, CI min = -0.011, CI max = 0.059, *p* > 0.05) insignificantly mediated the relationship between entertainment and repurchase intention. Similarly, brand awareness was found to insignificantly mediate the relationship between customisation and repurchase intention (*β* = -0.018, CI min = -0.048, CI max = 0.013, *p* > 0.05). On the other hand, brand awareness mediated the effects of trendiness (*β* = 0.212, CI min = 0.159, CI max = 0.267, *p* < 0.001), interaction (*β* = 0.212, CI min = 0.157, CI max = 0.266, *p* < 0.001), and word of mouth (*β* = 0.059, CI min = 0.023, CI max = 0.101, *p <* 0.001) on repurchase intention.

**Table 7:** Indirect effects.

Associations	Beta	CI–Min	CI—Max	*T*	*p*	Decision
ENT -> BAW -> RPI	0.023	-0.011	0.059	1.066	0.143	Reject
CUT-> BAW -> RPI	-0.018	-0.048	0.013	0.985	0.162	Reject
TRD-> BAW -> RPI	0.212	0.159	0.267	6.456	<0.001	Accept
INT-> BAW -> RPI	0.212	0.157	0.266	6.434	<0.001	Accept
WOM -> BAW -> RPI	0.059	0.023	0.101	2.509	0.006	Accept
ENT -> BIM -> RPI	-0.004	-0.022	0.016	0.303	0.381	Reject
CUT-> BIM -> RPI	0.024	0.005	0.047	1.862	0.031	Accept
TDR -> BIM -> RPI	0.080	0.045	0.120	3.507	<0.001	Accept
INT -> BIM -> RPI	0.048	0.017	0.085	2.311	0.010	Accept
WOM -> BIM -> RPI	0.046	0.021	0.077	2.672	0.004	Accept

**Note:** ENT: Entertainment; CUT: Customization; TDR: Trendiness; INT: Interaction; WOM: Word of Mouth; BAW: Brand Awareness; BIM: Brand Image; RPI: Repurchase Intention.

**Source:** Author’s data analysis.

The obtained results of the analysis further revealed that brand image (*β* = -0.004, CI min = -0.022, CI max = 0.016, *p* > 0.05) insignificantly mediated the relationship between entertainment and repurchase intention. Besides that, brand image was found to mediate the effects of customisation (*β* = 0.024, CI min = 0.005, CI max = 0.047, *p* < 0.05), trendiness (*β* = 0.080, CI min = 0.045, CI max = 0.120, *p* < 0.001), interaction (*β* = 0.048, CI min = 0.017, CI max = 0.085, *p* < 0.01), and word of mouth (*β* = 0.046, CI min = 0.021, CI max = 0.077, *p <* 0.01) on repurchase intention.

### 4.6. Artificial Neural Network (ANN) analysis

Three ANN models were employed in this study to evaluate the data. Model A consisted of five input constructs for brand awareness. Model B had five exogenous variables, and brand image served as the outcome variable. Lastly, Model C had brand awareness and image as input for repurchase intention. Table 8 depicts the results of ANN analysis. Overall, Model A, Model B, and Model C demonstrated high prediction accuracy, as both RMSE training and RMSE scores for testing were rather similar [90]. Apart from that, the results showed that all ANN models had good data fitting. The goodness of fit for Model A was 59%, while Model B recorded 62%. Model C recorded the highest goodness of fit at 68%.

**Table 8:** RMSE values of Artificial Neural Networks (N = 477).

	Model A (0.59)			Model B (0.62)			Model C (0.68)		
Network	RMSE (Training)	RMSE (Testing)	SSE (Testing)	RMSE (Training)	RMSE (Testing)	SSE (Testing)	RMSE (Training)	SSE (Testing)	SSE (Testing)
AAN1	0.351	0.319	0.898	0.547	0.489	1.383	0.418	0.353	1.072
AAN2	0.365	0.274	0.782	0.548	0.472	1.127	0.387	0.408	1.516
AAN3	0.335	0.352	0.824	0.500	0.582	1.750	0.398	0.392	1.255
AAN4	0.354	0.299	0.599	0.530	0.509	1.498	0.409	0.390	1.397
AAN5	0.343	0.317	0.868	0.484	0.655	1.701	0.369	0.469	1.350
AAN6	0.323	0.366	0.858	0.533	0.553	1.565	0.396	0.385	1.449
AAN7	0.383	0.276	0.779	0.554	0.454	1.033	0.378	0.450	1.267
AAN8	0.339	0.318	0.753	0.535	0.509	1.288	0.380	0.426	1.301
AAN9	0.306	0.431	0.936	0.536	0.525	1.415	0.395	0.390	1.084
AAN10	0.385	0.283	0.673	0.526	0.524	1.355	0.381	0.430	1.263
Mean	0.348	0.323	0.797	0.529	0.527	1.411	0.391	0.409	1.295
SD	0.024	0.048	0.103	0.021	0.058	0.229	0.015	0.034	0.142

**Source:** Author’s data analysis.

Sensitivity analysis was performed on all three ANN models to evaluate the contribution of each exogenous predictor for the endogenous constructs [89]. The results in Table 9 for Model A confirmed trendiness, word of mouth, and interaction as the most influential three factors that affect brand awareness. As for Model B, trendiness, interaction, and word of mouth were identified as three critical factors that meaningfully instigate brand image. For repurchase intention, brand awareness was the most significant factor, followed by brand image for high-tech products among Chinese consumers.

**Table 9:** Sensitivity analysis.

	Model A					Model B					Model C	
Network	ENT	CUT	TRD	INT	WOM	ENT	CUT	TRD	INT	WOM	BAW	BIM
ANN1	0.062	0.032	0.405	0.219	0.282	0.020	0.043	0.462	0.256	0.220	0.560	0.440
ANN2	0.055	0.028	0.391	0.280	0.246	0.042	0.053	0.384	0.268	0.253	0.544	0.456
ANN3	0.034	0.026	0.441	0.217	0.282	0.043	0.018	0.445	0.251	0.243	0.487	0.513
ANN4	0.046	0.047	0.425	0.228	0.254	0.025	0.005	0.479	0.283	0.208	0.489	0.511
ANN5	0.033	0.054	0.420	0.265	0.227	0.014	0.023	0.423	0.262	0.279	0.503	0.497
ANN6	0.080	0.027	0.420	0.220	0.253	0.049	0.018	0.479	0.280	0.179	0.532	0.468
ANN7	0.075	0.020	0.479	0.162	0.263	0.056	0.073	0.460	0.198	0.214	0.484	0.516
ANN8	0.051	0.019	0.445	0.208	0.276	0.034	0.025	0.463	0.279	0.199	0.504	0.496
ANN9	0.124	0.005	0.397	0.177	0.297	0.050	0.042	0.429	0.269	0.210	0.538	0.462
ANN10	0.112	0.105	0.436	0.088	0.259	0.040	0.021	0.437	0.328	0.174	0.502	0.498
Mean Importance	0.067	0.036	0.425	0.206	0.263	0.037	0.032	0.446	0.267	0.217	0.513	0.485
Relative Importance	16%	9%	100%	48%	61%	8%	7%	100%	60%	48%	100%	94%

**Note:** ENT: Entertainment; CUT: Customization; TDR: Trendiness; INT: Interaction; WOM: Word of Mouth; BAW: Brand Awareness; BIM: Brand Image; RPI: Repurchase Intention.

**Source:** Author’s data analysis.

The PLS-SEM results were compared with the outcomes of these ANN models. The results are tabulated in Table 10. The comparison depicts that the ranking of factors varies for Model A and Model B. However, the ranking of factors appeared the same for Model C.

**Table 10:** Comparision between PLS-SEM and ANN analysis.

PLS Path	Path coefficient	Normalized relative importance	Ranking based in PLS-SEM	Ranking based in ANN	Remark
Model A					
ENT-> BAW	0.042 (0.144)	16%	4	4	Match
CUT->BAW	-0.033 (0.163)	9%	5	5	Match
TRD->BAW	0.390 (<0.001)	100%	1	1	Match
INT->BAW	0.188 (<0.001)	48%	2	3	Mismatch
WOM->BAW	0.109 (0.0001)	61%	3	2	Mismatch
Model B					
ENT-> BIM	-0.014 (0.379)	8%	5	4	Mismatch
CUT->BIM	0.093 (0.021)	7%	4	5	Mismatch
TRD->BIM	0.312 (<0.001)	100%	2	1	Mismatch
INT->BIM	0.389 (<0.001)	60%	1	2	Mismatch
WOM->BIM	0.179 (<0.001)	48%	3	3	Match
Model C					
BAW->RPI	0.544 (<0.001)	100%	1	1	Match
BIM->PRI	0.255 (<0.001)	94%	2	2	Match

## 5. Discussion

This study provided evidence about the impact of SMMA on the repurchase intention held by Chinese high-tech consumers. The assessment was carried out on the five dimensions of SMMA (i.e., entertainment, customisation, trends, interaction, and WOM), two dimensions of brand equity (i.e., brand awareness and brand image), and one dimension of brand loyalty (i.e., repurchase intention). The research conclusively revealed the positive impact by entertainment and WOM on brand awareness in carrying out social media activities. This aligns with the outcomes of Seo and Park’s [24] study, which has indicated that social media marketing activities harness brand equity, creating brand awareness. Meanwhile, trendiness, interaction, and WOM positively impacted brand image, paralleling the results by Yadav and Rehman [25]. Therefore, WOM was explicitly associated with a significantly positive impact on brand awareness and image. Similarly, the work verified the hypothesis that brand awareness and brand image significantly impact customer loyalty. Thus, the current research fully supported and confirmed the SOR model.

Furthermore, the mediation effect obtained in this study depicted a satisfactory mediating effect between brand awareness and the factors of entertainment, WOM, and repurchase intention. Therefore, brand equity could be attributed as responsible for the relationship between the three factors. Besides, it yielded a sufficient mediating effect between trendiness, interaction, WOM, and repurchase intention, its weight for the relationship between brand image and all four factors. In contrast, brand image and brand awareness generated no mediating effect between customisation and repurchase intention.

### 5.1. Theoretical and practical implications

Social media marketing activities nurture and change consumer behaviour, which predictively influence their purchase and repurchase intention. The current study offered theoretical and practical implications. Most previous studies investigated the direct effects of social media marketing activities on purchase intention, but the current study successfully advanced the current literature by examining the mediating role of brand awareness and brand image in relation to repurchase intention of the brand offerings. The study’s findings would undoubtedly add value to the growing literature on social media marketing, brand equity, and repurchase intention. The current study utilised the SOR framework to justify the effects of social media marketing activities on brand awareness and brand image in relation to repurchase intention. Social media marketing activities have become powerful marketing strategies to meet consumers’ expectations and inclination to repurchase firms’ brand offerings. These marketing strategies are relevant to attract new consumers and significantly empower firms to manage customer relationships in order to retain the existing consumers.

The current study also offered three practical implications. Firstly, the study’s findings address firms’ management needs of developing and improving entertainment and customisation attributes in their social media marketing that can advance their efforts to harness brand equity, nurturing repurchase intention among technology product consumers [16]. Secondly, the current study emphasised repurchase intention as the function of customer loyalty. Social media marketing activities can significantly harness consumer-level of brand equity through brand awareness and brand image. All types of firms need to consider investing and building brand equity with the help of social media marketing activities. Currently, firms only concentrate on trendiness and the need to build social media marketing activities through entertainment, Interaction, customisation, and word of mouth. Lastly, the results of the current study confirmed the effects of social media marketing activities on brand awareness and brand image in relation to repurchase intention. Firms need to concentrate on building brand awareness and brand image with social media marketing activities. Consumers’ inclination to repurchase would develop with the right social media marketing activities, and marketers must harness brand-level equities to promote repurchase intention.

## 6. Limitation

However, this study has several limitations requiring further attention. For example, the components included in the current research model did not exhaustively list the explanatory variables possibly affecting SMMA. Moreover, the current research mainly focused on high-tech Chinese consumers, limiting the outcome generalisability across the market. Therefore, future works should look into designing research embedded with more variables to explore different social media’s effects on brand equity and brand loyalty across different brand-consumer segments. Besides, using the SOR-based model in this study might limit the research results. Thus, future researchers recommend confirming, replicating, or expanding the outcomes by integrating additional model constructs or using it across dissimilar cultural or geographic environments. This would deepen the scholarly understanding of customers’ repurchase intentions with more depth.

## Supporting information

S1 Data (CSV)
